# Integrated geochemical and PAH analysis with benthic foraminifera to assess environmental quality in a tropical estuary

**DOI:** 10.1007/s10661-026-15676-5

**Published:** 2026-07-14

**Authors:** Luanna Maia Carneiro, Ruth Souza dos Santos Rocha, Simone Souza de Moraes, Gilson Barbosa Dourado, José Marques Lopes, Gisele Mara Hadlich, Taise Bomfim de Jesus

**Affiliations:** 1 Programa de Pós-Graduação Em Geoquímica: Petróleo E Meio Ambiente, Instituto de Geociências, Universidade Federal da Bahia, Salvador, Brazil; 2 Departamento de Ciências Humanas E Tecnologias (Campus XIX), Universidade Do Estado da Bahia, Camaçari, Brazil; 3 Laboratório de Detecção E Instrumentação Nuclear (LDIN-SE/7), Instituto Militar de Engenharia, Rio de Janeiro, Brazil; 4 Departamento de Ciências Exatas, Universidade Estadual de Feira de Santana, Novo Horizonte, Feira de Santana, Bahia Brazil

**Keywords:** Sediment, Chemical elements, Foraminifera, Monitoring, Tropical estuary

## Abstract

This study assessed the environmental quality of the Serinhaém River estuary (Bahia, Brazil). The characterization was based on concentrations of chemical elements, total organic carbon (TOC), physicochemical parameters, and polycyclic aromatic hydrocarbons (PAHs) in surface sediments, combined with benthic foraminifera as bioindicators. Samples were collected at multiple sites during two seasonal campaigns conducted in 2013 and 2022, representing pre- and post-spill conditions. Chemical element concentrations showed no significant temporal differences but high spatial variability, with Fe, Al, and Mn predominating, indicating association with oxyhydroxides and aluminosilicates. PAHs in 2022 indicated low-to-moderate contamination, predominantly of pyrogenic origin, and did not exceed threshold effect levels (TEL and PEL). In 2013, benthic foraminiferal distribution was mainly influenced by precipitation, promoting transport toward the estuary mouth. In 2022, salinity and pH became the dominant controlling factors. Significant associations were observed between PAHs and benthic foraminiferal assemblages. Negative associations were observed between naphthalene and anthracene and the diversity and abundance of tolerant species (e.g., *Ammonia beccarii* and *Textularia gramen*), while dibenzo[a,h]anthracene showed a positive association with *Ammoglobigerina globigeriniformis*. Overall, the estuary remains preserved regarding elemental concentrations; however, PAHs influence benthic assemblages, highlighting foraminifera as effective indicators of environmental quality and hydrocarbon contamination.

## Introduction

Over recent decades, estuarine ecosystems have been subjected to increasing anthropogenic pressures that disrupt their natural variability, rendering them particularly vulnerable to contamination (Martínez-Colón et al., 2018). Within these dynamic systems, sediment quality is widely recognized as a sensitive and reliable indicator, often referred to as a geomarker, of environmental pollution (Aduwo & Adeniyi, 2018; Chuan & Yunus, 2019). Acting as major sinks for contaminants such as trace elements and polycyclic aromatic hydrocarbons (PAHs), sediments typically exhibit higher contaminant concentrations than the overlying water column, thereby preserving records of pollution sources and transport pathways over time (Frontalini & Coccioni, 2011; Oron et al., 2021).

In addition to geochemical approaches, benthic foraminifera have been extensively employed as effective bioindicators in environmental assessments of estuarine and coastal systems (Balachandar et al., 2023; Martins et al., 2019). Their suitability is associated with several advantageous traits, including ease of sampling, high sedimentary abundance, low mobility, and rapid response to environmental changes due to short life cycles (Baptista Neto et al., 2017; Price et al., 2019). These organisms are particularly sensitive to variations in physicochemical parameters and contaminant levels (A’ziz et al., 2021; Balachandar et al., 2023; Gómez-León et al., 2018; Young et al., 2021).

Environmental stress is often reflected in morphological abnormalities, which have been widely documented in coastal environments impacted by contamination from trace elements, hydrocarbons, or eutrophication (Balachandar et al., 2023; Buosi et al., 2010; Luciani, 2007; Ruiz et al., 2012). Typical responses of benthic foraminiferal assemblages to pollution include reduced diversity, increased dominance of tolerant or opportunistic species, and alterations in morphology and reproductive patterns (Lei et al., 2015). Their persistence in impacted environments—where they are often among the last eukaryotic organisms to disappear—further reinforces their value as robust indicators of environmental degradation (Alve, 1991; Martínez-Colón et al., 2018).

Importantly, foraminifera record environmental changes within their tests, enabling the reconstruction of past environmental conditions from sub-recent sediments and fossil archives (Baptista Neto et al., 2017; Frontalini & Coccioni, 2011). This feature is crucial for assessing recent environmental changes and anthropogenic impacts through the establishment of baseline contaminant levels against which subsequent disturbances can be evaluated (Baptista Neto et al., 2017; Oron et al., 2021).

In 2019, a large-scale oil spill impacted the Brazilian coastline (Lourenço et al., 2020; Oliveira et al., 2020), causing significant environmental and socioeconomic damage (Araújo et al., 2020). According to the Brazilian Institute of the Environment and Renewable Natural Resources (IBAMA), petroleum residues reached the Serinhaém River estuary (IBAMA, 2020), an area of high ecological relevance located within the Pratigi Environmental Protection Area (EPA).

Despite the widespread application of benthic foraminifera and sediment geochemistry in estuarine environmental assessments, studies specifically addressing oil spill impacts using this bioindicator remain limited. Moreover, no previous investigation has employed benthic foraminifera as bioindicators in the Serinhaém River estuary. Data collected in 2013 provide a unique pre-spill environmental baseline, enabling a robust comparison with post-spill conditions following the 2019 event.

Integrated studies combining benthic foraminifera, chemical elements, and PAHs in estuarine sediments remain relatively scarce, particularly within protected areas. To date, this integrated approach has not been applied to the Serinhaém estuary, highlighting a knowledge gap regarding its biological and geochemical responses to petroleum contamination. Therefore, this study aims to assess the environmental quality of the Serinhaém River estuary following the 2019 oil spill through an integrated analysis of the distribution, diversity, and abundance of benthic foraminifera, sedimentary chemical element concentrations measured in 2013 and 2022, and PAH levels determined in 2022.

## Material and methods

### Study area

**Fig. 1:**
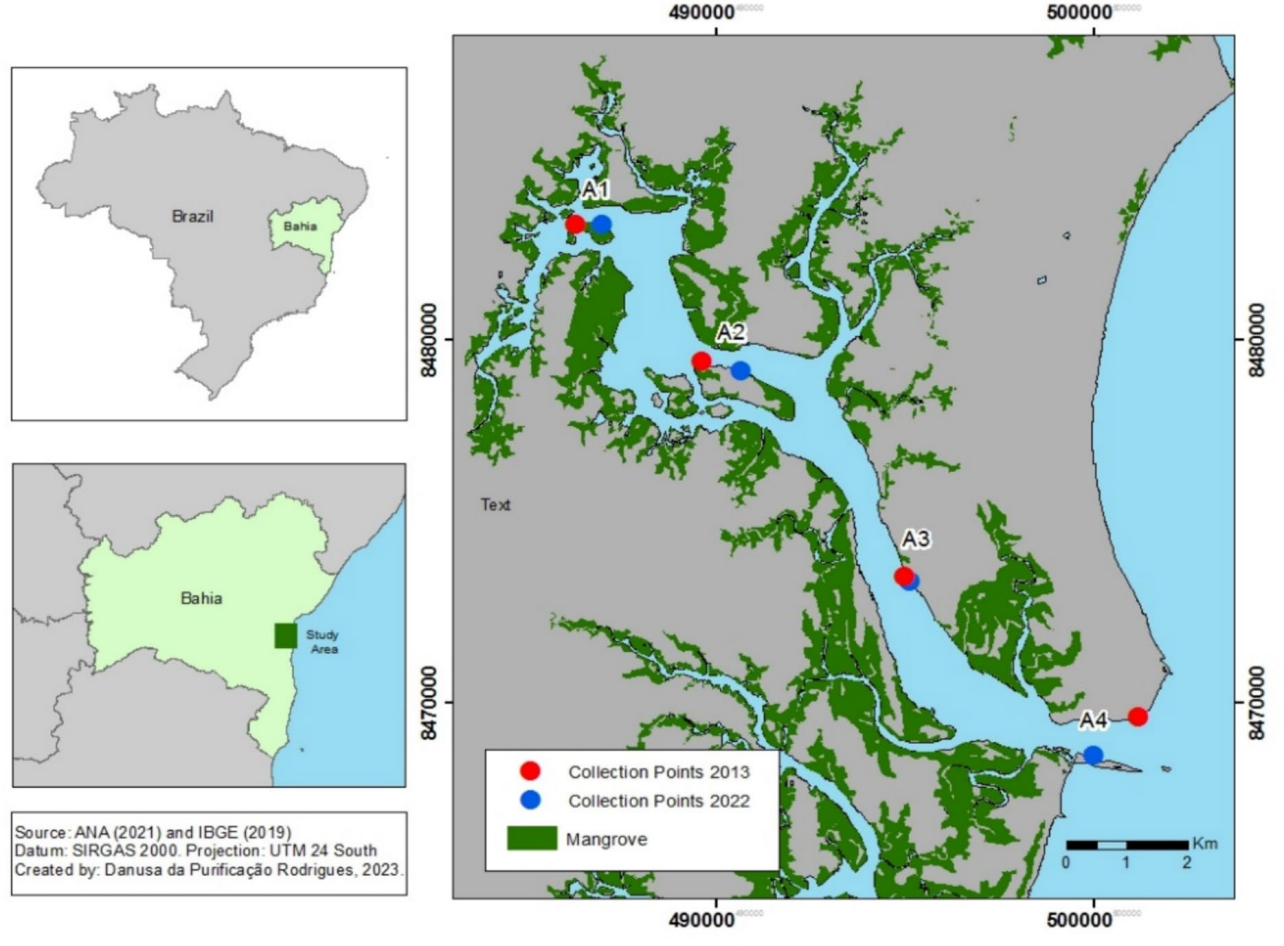
Collection points for analysis of benthic foraminiferal fauna in 2013 and 2022 in the Serinhaém river estuary, Bahia

The Serinhaém River estuary is part of the Pratigi EPA (Fig. 1), one of the highest-priority regions for biodiversity conservation within the Central Atlantic Forest Corridor in South America. This status is due to the high diversity of mammals, invertebrates, birds, reptiles, and amphibians (OCT, 2015). The region is characterized by a tropical rainforest climate with no dry season, presenting average monthly rainfall exceeding 60 mm and annual precipitation above 1500 mm (Bahia, 2014).

This estuary also constitutes the northernmost portion of Camamu Bay, the second largest bay in the state (Santos & Nolasco, 2017), functioning as a sub-estuary (Dominguez, 2010). It forms a dynamic estuarine system influenced by both fluvial and marine processes, ultimately draining into Camamu Bay and connecting to the Atlantic Ocean along an approximate 30 km (Santos & Nolasco, 2017). Additionally, it is a shallow system (Hatje et al., 2008), with an average depth of approximately 7.3 m (Affe et al., 2018), and exhibits sedimentary features typical of coastal environments, including mangroves, vegetated fluvial islands, muddy banks, sandy shoals, tidal flats, and bars (OCT, 2015). The area is relatively well preserved with respect to chemical element contamination (Carneiro et al., 2021a), and its organic matter predominantly originates from higher plants exhibiting a C3 photosynthetic pathway (Carneiro et al., 2021b).

Surface sediment samples were collected at four sites (Fig. 1) in April 2013 and July 2022, both corresponding to the rainy season in the study area. The selection of sampling sites in 2022 was based on the locations established during the 2013 campaign. Following the 2019 oil spill accident, oil slicks were observed along the northeastern coast of Brazil, including within the estuary and on adjacent beaches.

### Sample collection and physicochemical characterization

Samples were collected using a stainless-steel Van Veen dredge, stored in plastic containers, and kept refrigerated until analysis. For this study, only the upper 5 cm of sediment were retained and separated using a decontaminated glass rod. The parameters pH, dissolved oxygen (DO), and salinity were measured in situ at the water surface using a multiparameter probe (AKSO, model 88). Both sediment and water samples were collected at low tide.

Sediment samples were freeze-dried (LIOTOP, model L101) prior to all analyses. Particle size analysis followed the methodology described by EMBRAPA (1997), with adaptations according to Carneiro et al. (2021a). Total organic carbon (TOC) and total nitrogen (TN) were determined using the U.S. Environmental Protection Agency method USEPA-NCE (2002), with modifications described in Carneiro et al. (2021b). Briefly, 0.1 g of decarbonated sediment (inorganic carbon removed using hydrochloric acid, HCl) was weighed into a tin capsule using an analytical balance. TOC and TN concentrations were then measured using an elemental analyzer (LECO, model CNH 628).

### Chemical element concentrations

The concentrations of chemical elements (Al, Fe, As, Ba, Pb, Co, Cd, Cr, Mn, Ni, V, and Zn) in the sediment were determined by partial extraction following an adaptation of the U.S. Environmental Protection Agency method USEPA-3051A (2007). Freeze-dried samples were ground using an agate ball mill (RETSCH, model PM400) to ensure homogenization, and at least 10 g of sediment was processed. Subsequently, 0.5 g of the homogenized sample was accurately weighed and transferred to digestion vessels, followed by the addition of 1 mL of HCl and 3 mL of HNO₃. The samples were digested in a microwave system (Anton Paar, model Multiwave GO Plus) at 180 °C for 1 h, with 5-min heating and cooling ramps (total ramp time of 10 min).

The samples were filtered and transferred to a volumetric flask, followed by dilution to 50 mL with ultrapure water. Elemental concentrations were determined by inductively coupled plasma optical emission spectrometry (ICP-OES). The instrumental parameters used for ICP-OES analysis are presented in Table 1.

**Table 1:** Parameters of the ICP-OES used to determine chemical elements

Parameters	Characteristics
Misting chamber	Cyclonic (single pass)
Nebulizer	OneNeb
Capacity (kW)	1.2
Plasma gas flow (L min^−1^)	15
Auxiliary gas flow (L min^−1^)	1.5
Gas flow nebulization (L min^−1^)	0.75
Elements and wavelengths (nm)	Ba (493.408); Cd (228.802);
	Co (228.615); Cr (267.716);
	As (188.980); Mn (257.610);
	Ni (231.604); Al (308.215)
	Pb (220.353); V (311.837);
	Zn (213.857); Fe (259.940);
Quantification limits (mg/kq)	Ba (5.0); Cd (1.0); Co (1.0); Cr (1.0); As (5.0); Mn (5.0); Ni (1.0); Al (5.0); Pb (2.0); V (5.0); Zn (1.0); Fe (5.0)

To ensure analytical reliability, the certified sediment reference material STSD-3 was used. A procedural blank was included in each analytical batch, and 30% of the samples were prepared in triplicate. Element concentrations were compared with the Threshold Effect Level (TEL), Probable Effect Level (PEL), and Effects Range-Low (ERL) guidelines proposed by Macdonald (1996), except for Al, Fe, Ba, Mn, and Co, for which no corresponding values were provided. Chemical element recoveries and the TEL, PEL, and ERL guideline values are presented in Table 2.

**Table 2:** Recovery percentage of certified reference material (STSD-3)

Element	Al	Fe	As	Ba	Cd	Co	Mn	Ni	Pb	Zn
Rec perc %	*	70	114	*	100	102	85.61	78.16	93.79	90.73
ERL	-	-	8.2	-	1.2	-	-	20.9	46.7	150
TEL	-	-	7.24		0.68	-	-	15.9	30.2	124
PEL	-	-	41.6	-	4.21	-	-	42.8	112	241

*There are no values for partial concentration for the elements Al and Ba

### Enrichment factor

To evaluate the intensity of elemental concentrations and distinguish between natural and anthropogenic origins in the Serinhaém River estuary, the enrichment factor (EF) was calculated. Aluminum was selected as the geogenic normalizer to compensate for variations in grain size and mineralogy. The baseline values (background) were established using the mean concentrations obtained in the 2013 sampling campaign. The EF for each chemical element was calculated according to the following Eq. (1) (Taylor & McLennan, 1985; Sutherland et al., 2000; Indraswari et a., 2024):

1 $$EF=\frac{\left(C_x/C_{Al}\right)sample}{\left(C_x/C_{Al}\right)2013}$$

where ${({C}_{x}/{C}_{Al})}_{sample}$ is the ratio between the concentration of the target element and aluminum in a given sample from the 2022 campaign, and ${({C}_{x}/{C}_{Al})}_{2013}$ is the average ratio of the same elements calculated for the 2013 baseline. EF values close to or below 1.5 indicate a predominantly natural source, whereas EF values greater than 1.5 suggest an anthropogenic contribution or enrichment.

Aluminum was selected as the reference element because it is a major constituent of aluminosilicate minerals and exhibits relatively conservative behavior in sediments, being largely insensitive to redox-driven mobility and most diagenetic processes. Normalization to Al compensates for grain-size and mineralogical variability, reducing quartz dilution effects associated with sandy fractions and minimizing natural hydrodynamic influences on sediment composition. This approach enhances the discrimination between natural geochemical variability and potential anthropogenic metal enrichment.

### PAH determination

For PAH determination, samples were collected and transferred to aluminum trays previously calcined at 450 °C for 4 h to eliminate organic residues. To minimize the risk of cross-contamination, all laboratory materials were decontaminated prior to use by immersion in a 5% Extran® solution for 12 h, followed by thorough rinsing with distilled water. After complete drying, a final cleaning step was performed using dichloromethane (DCM).

In this study, only the 16 PAHs classified as priority pollutants by the USEPA-3550C were analyzed. For PAH extraction, n-hexane (HPLC grade, Merck, Germany) and dichloromethane (HPLC grade, Merck, Germany) were used, while sodium sulfate (Na₂SO₄; P.A., Merck, Germany) was employed to remove residual moisture. Certified reference materials included the internal standard p-terphenyl-d14 (pTer-d14) (AccuStandard; dissolved in dichloromethane, 2 mg·L⁻^1^); a mixed PAH standard solution containing naphthalene (Nap), acenaphthylene (Acy), acenaphthene (Ace), fluorene (Flu), phenanthrene (Phe), anthracene (Ant), fluoranthene (Fla), pyrene (Pyr), benzo[a]anthracene (BaA), chrysene (Chr), benzo[b]fluoranthene (BbF), benzo[k]fluoranthene (BkF), benzo[a]pyrene (BaP), indeno[1,2,3-c,d]pyrene (IP), dibenzo[a,h]anthracene (DahA), and benzo[g,h,i]perylene (BghiP) (AccuStandard; dissolved in dichloromethane:benzene, 1:1, 2.0 mg·mL⁻^1^); and a mixture of deuterated internal standards comprising naphthalene-d8 (Nap-d8), acenaphthene-d10 (Acen-d10), and chrysene-d12 (Chr-d12) (AccuStandard; in dichloromethane, 4000 μg·mL⁻^1^).

Sediment samples were freeze-dried for 105 h using a lyophilizer (LIOTOP L101, Brazil). PAH extraction followed the USEPA  (2007) ultrasonic extraction protocol (Method 3550 C), as described in Carneiro et al. (2026).

PAH determination was performed by gas chromatography coupled with mass spectrometry (GC–MS). Analyses were conducted using a 7890 A gas chromatograph equipped with a 7683B autosampler and coupled to a 5975 C mass selective detector (Agilent Technologies). Separation was achieved using a DB-5-ms capillary column (5% diphenylpolymethylsiloxane; 30 m × 0.25 mm ID × 0.25 μm film thickness). Helium was used as the carrier gas at a flow rate of 1 mL·min⁻^1^. Injections were performed in splitless mode at 300 °C with an injection volume of 1 μL. The oven temperature program was as follows: initial temperature of 50 °C (held for 1 min), increased at 20 °C·min⁻^1^ to 200 °C, then at 10 °C·min⁻^1^ to 260 °C (held for 3 min), followed by a ramp of 2.5 °C·min⁻^1^ to 300 °C with a final hold of 8.5 min. The transfer line temperature was maintained at 300 °C. Analyses were conducted in selected-ion monitoring (SIM) mode to enhance sensitivity and selectivity. Calibration curves were prepared in dichloromethane over the concentration range of 5.0 to 200.0 μg·L⁻^1^.

Procedural blanks were included in each analytical batch. Limits of detection (LD) and quantification (LQ) (Table 3) were determined based on calibration curve parameters using the equations LD = 3.3 × (s/S) and LQ = 10 × (s/S), where s represents the standard deviation and S the slope of the calibration curve. The standard deviation was calculated from seven replicates of the lowest calibration level. For statistical analyses, concentrations below the LQ were assigned a value equal to half of the respective LQ. The LD values ranged from 0.27 ng·g⁻^1^ for BaA to 0.45 ng·g⁻^1^ for Ace. Recovery of the internal standard p-terphenyl-d14 was considered acceptable within the range of 80 to 110%, in accordance with Ribani et al. (2004).

**Table 3:** Limits of quantification (LQ) for PAHs in sediment samples from the Serinhaém River estuary

	Abbreviations	Full name	LQ (ng g^−1^)
Low molecular weight (LMM)	NaP	Naphthalene	1.09
	Acy	Acenaphthylene	1.11
	Ace	Acenaphthene	1.35
	Flu	Fluorene	0.98
	Phe	Phenanthrene	1.17
	Ant	Anthracene	1.15
High molecular weight (HMW)	Fla	Fluoranthene	1.27
	Pyr	Pyrene	1.04
	Chr	Chrysene	1.22
	BaA	Benzo[a]anthracene	0.81
	BaP	Benzo[a]pyrene	1.19
	BbF	Benzo[b]fluoranthene	0.84
	BkF	Benzo[k]fluoranthene	1.19
	DahA	Dibenzo[a,h] nthracene	1.18
	BghiP	Benzo[g,h,i]perylene	1.20
	IP	Indeno[1,2,3-cd]pyrene	1.17

### Diagnostic ratios

To distinguish between petrogenic and pyrogenic sources of PAHs in environmental samples, diagnostic ratios based on specific isomer pairs were applied (Budzinski et al., 1997; Chen & Chen, 2011; Santos et al., 2017; Yunker et al., 2002). The validity of this approach is based on differences in the thermodynamic stability of PAH isomers (Santos et al., 2017).

In general, petroleum-derived contamination is associated with a predominance of low-molecular-weight (LMW) PAHs (two to three aromatic rings), whereas combustion-related sources, such as coal burning and vehicular emissions, are characterized by high-molecular-weight (HMW) PAHs (four to six rings) (Lia et al., 2024). Accordingly, diagnostic ratios such as Ant/(Ant + Phe), Fla/(Fla + Pyr), and ΣLMW/ΣHMW PAHs (Yunker et al., 2002) are widely used to assess PAH sources across different environmental contexts (Chen & Chen, 2011; Guimarães et al., 2020; Qiao et al., 2006; Yunker et al., 2002; Zhang et al., 2021, 2024).

### Foraminiferal analysis

Sediment samples were washed under running water through a 0.062-mm mesh sieve and dried in an oven at 60 °C for foraminiferal analysis. Three grams of dry sediment from each sample were transferred to beakers and processed under a fume hood to separate foraminiferal tests from the sediment by flotation in trichloroethylene (Scott et al., 2007).

All recovered tests (total assemblage) were examined under a stereomicroscope (Olympus models SZ2-LGB and SZ40), mounted on micropaleontological slides, and identified to the species level using specialized literature. During identification, the taphonomic characteristics of the tests (color, degree of wear, and presence of pyrite) were also evaluated, following the criteria proposed by Moraes and Machado (2003).

Species were classified according to their relative frequency as: principal components (> 5%), accessory components (1–4.9%), and trace components (< 1%). Test preservation was assessed based on color, white (B), yellow (Y), brown (Br), mottled (Mo), and black (Bk), and type of wear: normal (N), abrasion (A), dissolution (D), breakage (B), and mixed (Mi). The tests were also classified according to wall type and the presence of pyrite.

### Shannon–Wiener Index (H’)

The Shannon–Wiener index measures the uncertainty in predicting the species identity of an individual randomly selected from a sample containing S species and N individuals (Uramoto et al., 2005). Thus, higher uncertainty corresponds to higher index values and, consequently, greater diversity.

This index is based on two assumptions: (1) individuals are randomly sampled from an effectively infinite population and (2) all species are represented in the sample. The formula for this index is shown in Eq. (2):

2 $$H^\backprime=-\overset S{\underset{i=l}{\sum\;}}\;p_i\cdot\ln\ln\;p_i$$

In this equation, *pᵢ* represents the proportion of the *i-th* species in the sample. The logarithmic base may be 2, 10, or e; in this study, base 10 was adopted. The Shannon index ranges from 0 to a maximum value, reaching zero when only one species is present. It attains its maximum when all species have equal abundances. This index is sensitive to species richness and evenness, but tends to give more weight to rare species due to the logarithmic component.

### Pielou’s Evenness Index (J)

Pielou’s evenness index is derived from the Shannon–Wiener index and describes how evenly individuals are distributed among species in a community. The formula for this index is shown in Eq. (3):

3 $$J=\frac{H^\prime}{H_{max}}=\frac{H^\prime}{\ln\ln\;S}$$

where H’ is the Shannon–Wiener index and *Hₘₐₓ* represents the maximum possible diversity, which occurs when all species have equal abundances. *Hₘₐₓ* is calculated as the natural logarithm (ln) of species richness (S). Values of *J* range from 0 to 1. When all species have equal abundances, *J* = 1, indicating maximum evenness. As dominance by one or a few species increases, *J* approaches zero.

### Margalef index

The Margalef index demonstrates specific wealth and refers to the total number of individuals. It is used to estimate diversity based on the numerical distribution of individuals or species. The higher the index value, the greater the diversity of the sample. It is estimated by the Eq. (4):

4 $$D_{Mg}=\frac{S-1}{\ln\ln\;\left(N\right)}$$

where *S* is the total number of species, *N* is the total number of individuals, and *ln* denotes the natural logarithm. The Margalef index has no fixed upper limit, and its interpretation is comparative, with higher values indicating greater richness.

The data were statistically analyzed using the R software. Nonparametric methods were applied, including tests for equality of distributions and the Mann–Whitney test.

## Results and discussion

### Geochemical parameters

Tables 4–6 contain general data and descriptive statistics for pH, particle size, TOC, TN, salinity, DO, temperature, and chemical elements from 2013 and 2022. In the sampling carried out in 2013, the pH values of the water did not vary significantly, with the highest alkalinity in area 4 (A4) (8.37) due to its location downstream and, consequently, under greater marine influence. This is corroborated by the fact that the most distant points had the lowest alkalinity (A2 with 7.90). In the sampling carried out in 2022, there was also a slight variation in water pH between collection points, and an increase in basicity was recorded towards the mouth of the estuary. The pH values were 7.77, 7.99, 8.12, and 8.42 for A1, A2, A3, and A4, respectively.

**Table 4:** Values of physical–chemical variables and chemical elements in the sediment Serinhaém River estuary in the 2013 and 2022 campaigns

Areas	Year	DO	pH	Salinity	Mud	TOC	As	Ba	Co	Cr	Al	Fe	Mn	Ni	Pb	V	Zn	Cu
A3	2013	3.72	8.02	29.75	1.61	< LQ	9.20	48.20	< LQ	40.70	25,826	15,480	115	111	48.20	24.98	33.37	3.40
A4	2013	4.42	8.37	35.43	1.87	< LQ	< LQ	16.80	< LQ	13.10	8745	8933	51.60	< LQ	< LQ	6.62	15.44	< LQ
A1	2022	*	7.77	21.90	58.23	5.23	12.91	77.37	8.11	51.48	34,575	21,908	177	8.21	12.47	2.9	50.61	10.01
A2	2022	7.80	7.99	25.90	22.77	0.28	2.50	26.98	0.50	5.00	7851	5374	76.69	< LQ	3.45	2.4	12.11	1.83
A3	2022	8.40	8.12	30.50	80.87	1.79	9.15	100	1.76	21.12	19,220	12,226	129	1.51	10.73	35.8	19.72	4.57
A4	2022	10.50	8.42	34.50	71.49	< LQ	< LQ	23.93	< LQ	1.13	2854	3187	79.92	< LQ	3.17	13.8	6.70	< LQ

*Not measured

Mud values are the sum of silt and clay

Units of measurement: DO (Dissolved oxygen) (mg L−1); mud, sand, and TOC (%)

Values of chemical elements in mg kg.−^1^

< LQ indicates below quantification values: TOC LQ = 0.04%. Cd < LQ (2013 and 2022)

As expected, salinity values decreased upstream during the 2013 campaign, reflecting the transition from marine-dominated to freshwater-dominated conditions. In the 2022 campaign, salinity showed the same behavior, increasing towards the mouth. The DO (mg/L) in the 2013 campaign presented its lowest value in A3 (3.72 mg/L) and the highest in A2 (4.72 mg/L); however, in the 2022 campaign, DO values increased towards the mouth (7.80, 8.40, and 10.50 mg/L for A2, A3, and A4, respectively).

The particle size of the sediments in both campaigns was classified into sand and mud (silt + clay), showing different behaviors between them. In the 2013 campaign, the sand fraction was predominant downstream, and only A2 presented a significant amount of mud (67.37%). In 2022, the relationship was reversed, with a predominance of mud downstream, while in A2, the sand fraction content was approximately 80%. The predominance of sand observed in the 2013 campaign aligns with previous studies carried out in the Serinhaém River estuary (Carneiro et al., 2021a; Santos & Nolasco, 2017). However, the high mud content indicates the prevalence of low hydrodynamic energy conditions in the 2022 sampling.

In the 2013 campaign, the highest TOC was recorded in A1 (1.47%), followed by A2 (0.46%), with values below the quantification limit (LOQ = 0.04%) in the other areas. In the 2022 campaign, the values varied considerably, being higher in A1 (5.23%) than in A2 (0.28%), A3 (1.79%), and A4 (below the LQ). A1 is the most upstream collection point of the estuary; therefore, the water residence time tends to be longer at this location (Al-Enezi et al., 2022). It is the closest to the city of Ituberá, which justifies the increase in organic load due to sewage discharge and waste disposal, consequently contributing to the retention of chemical elements in the sediment (Carneiro et al., 2021a) such as Cu and Zn in 2022.

The descriptive measures (Table 4) highlight the difference in physicochemical parameters and particle size between the years analyzed (2013 and 2022). Regarding the averages, there is no significant variation in physicochemical parameters and particle size between the years, except for DO, % mud, and % sand. Regarding variability, % mud (2013) and % TOC (2013, 2022) present high variability (coefficient of variation > 0.05).

The Mann–Whitney test was performed to check whether changes in physicochemical parameters and particle size occurred over time. According to the results in Table 5, the differences in the means of DO, %mud, %sand, and TOC are not statistically significant (α = 0.05). This result suggests that no significant changes occurred in the sedimentological characteristics and oxygenation conditions of the environment between the years evaluated.

**Table 5:** Descriptive measurements and Mann–Whitney test (*p* < 0.05) for physicochemical and particle size parameters

Parameters	Year	Average	min–max	Standard deviation	*p*-value
DO (mg L^−1^)	2013	4.21	3.72–4.72	0.45	0.057
	2022	8.90	7.80–10.50	1.42	
pH	2013	8.05	7.90–8.37	0.22	0.886
	2022	8.08	7.77–8.42	0.27	
Salinity	2013	29.72	25.90–35.40	4.12	0.886
	2022	28.20	21.90–34.50	5.48	
Mud (%)	2013	19.37	1.61–67.40	32.08	0.114
	2022	58.34	22.8–80.90	25.47	
Sand (%)	2013	80.64	32.60–98.40	32.08	0.114
	2022	41.22	19.10–77.20	25.48	
TOC (%)	2013	0.49	0.02–1.47	0.68	0.460
	2022	1.83	0.02–5.23	2.40	

### Chemical elements

In Table 6, the average concentrations of chemical elements in 2013 followed the order: Al > Fe > Mn > Ni > Ba > Zn > Cr > V > Pb > As > Cu > Cd > Co.

**Table 6:** Descriptive statistics for chemical element concentrations by sampling year and *p*-values for the Mann–Whitney *U*-test (*p* < 0.050)

Elements chemicals	Year	Average	min–max	Stand deviation	*p*-value Mann–Whitney *U*-test	*EF*
Al	2013	9346.35	1076.2–25,826.3	11,521.572	0.486	-
	2022	16,125.645	2854.7–34,575.7	14,077.790		
As	2013	4.175	2.50–9.20	3.350	0.620	0.94
	2022	6.765	2.50–12.91	5.158		
Ba	2013	18.325	2.50–48.20	20.834	0.114	1.92
	2022	57.133	23.93–100.26	37.777		
Cd	2013	0.500	0.50–0.50	0.000	-	0.32
	2022	0.500	0.50–0.50	0.000		
Co	2013	0.500	0.50–0.50	0.000	-	5.43
	2022	2.717	0.50–8.11	3.644		
Cr	2013	14.400	0.50–40.70	18.347	0.686	0.25
	2022	19.683	1.13–51.48	22.898		
Cu	2013	1.225	0.50–3.40	1.450	0.219	2.53
	2022	4.228	0.50–10.01	4.211		
Fe	2013	7387.525	2426.6–15,480.8	6174.980	0.486	0.42
	2022	10,674.333	3187.6–21,908.3	8421.254		
Mn	2013	63.163	26.65–115.20	37.374	0.114	1.27
	2022	115.910	76.69–117.44	47.633		
Ni	2013	29.250	0.50–111.00	54.541	0.878	0.05
	2022	2.681	0.50–8.21	3.718		
Pb	2013	12.800	1.00–48.20	23.600	0.301	6.35
	2022	7.45	3.17–12.47	4.839		
V	2013	13.725	2.40–35.80	15.628	0.486	3.21
	2022	25.650	6.62–58.62	23.276		
Zn	2013	15.715	6.70–33.37	12.423	0.886	0.76
	2022	22.285	6.70–50.61	19.625		

In the 2022 campaign, the average concentrations were presented in the following order: Al > Fe > Mn > Ba > V > Zn > Cr > Pb > As > Cu > Co > Ni > Cd. The high concentrations of Al, Fe, and Mn were expected since, according to Santos and Nolasco (2017), the Serinhaém River estuary is located in the Camamu Basin, which consists mainly of sandstones rich in Fe and Mn oxy-hydroxides. The high concentrations of these three elements are consistent with values found in previous studies in the area (Carneiro et al., 2021a; Hatje et al., 2008; Pereira et al., 2022).

In the 2013 campaign, Cd and Co were found below the LQ (1.0 mg kg⁻^1^) in all samples. In 2022, only Cd concentrations exhibited this behavior, which is consistent with the values found by Carneiro et al. (2021a) in vertical sediment profiles within the estuary.

In both 2013 and 2022, Ba presented a high average value, with its highest concentration in A3. Concentrations increased from 2013 to 2022; in 2013, values ranged from 2.5 in A2 to 48.2 in A3, with an average of 18.3 mg kg⁻^1^. In 2022, values varied from 23.93 in A4 to 100.26 in A3, averaging 57.13 mg kg⁻^1^. The high values were expected due to the proximity to the Camamu Bay, which is enriched in Ba; furthermore, previous studies have also emphasized the high values found in this area (Carneiro et al., 2021a; Hatje et al., 2008; Pereira et al., 2022).

In the 2013 campaign, A3 was the area with the highest concentration of all elements, except for those below the LQ. In 2022, all chemical elements showed the highest concentrations in A1, the closest point to the city of Ituberá, followed by A3. The lowest concentrations were found in A4 and A2, respectively. It is worth noting that A1 also exhibited the highest TOC.

The behavior of chemical elements in both campaigns generally follows that of Al, Fe, and Mn. The points with the highest values for these three elements also presented the highest levels of the others, suggesting that these chemical elements may be associated to Fe and Mn oxyhydroxides, as well as aluminosilicates (Carneiro et al., 2021a). Although most elements follow the spatial distribution of lithogenic tracers, enrichment factors indicate the enrichment of specific elements relative to the levels expected from a predominantly natural origin.

Elements presenting EF values below or close to the 1.5 threshold are generally considered to exhibit little or no significant enrichment relative to natural background levels, suggesting a predominance of geogenic controls. In the Serinhaém River estuary, this pattern was observed for As (0.94), Cd (0.32), Cr (0.25), Fe (0.42), Mn (1.27), Ni (0.05), and Zn (0.76). The low EF values indicate that the concentrations of these elements are largely consistent with the natural geochemical composition of the estuarine sediments and are likely influenced by sedimentary processes controlling the distribution of aluminosilicates and associated mineral phases. Overall, these results suggest that the lithogenic geochemical framework of the estuary remains largely preserved for most of the evaluated elements.

In contrast, EF values above 1.5 were recorded for Ba (1.92), Cu (2.53), V (3.21), Co (5.43), and Pb (6.35), indicating enrichment relative to the local geochemical background and suggesting the influence of additional sources or retention mechanisms. The moderate enrichment observed for Ba may be associated with regional geological characteristics, including the historical occurrence of barite deposits and mining activities in the Camamu Basin region. Likewise, the enrichment of Cu and Pb may reflect contributions from localized anthropogenic activities, such as urban runoff, domestic effluents, small-vessel traffic, or other human pressures operating within the watershed. The elevated EF values of V and Co indicate a distinct geochemical behavior compared with the predominantly lithogenic elements, although the specific sources of these enrichments cannot be conclusively identified based solely on EF data. Nevertheless, the enrichment of V is noteworthy because this element is commonly associated with petroleum-bearing materials and petroleum-derived residues. In this context, its occurrence may be consistent with hydrocarbon-related inputs previously reported for the region, including those potentially linked to the 2019 oil spill that affected large portions of the northeastern Brazilian coastline. Taken together, these findings suggest that, although the estuary retains its overall natural geochemical character, selected elements exhibit enrichment patterns compatible with localized anthropogenic influences and possible hydrocarbon-related contributions that have become incorporated into the recent sedimentary record.

No significant differences were found when comparing the pseudo-total concentrations between the two sampling periods. However, the elements showed different spatial behaviors between the two campaigns regarding the collection areas, as evidenced above.

To place these findings into a regional context, the elemental concentrations obtained in the present study were compared with those reported in previous investigations conducted in the Serinhaém estuary and other estuarine environments. Previous studies conducted in the Serinhaém estuary, particularly those of Carneiro et al., (2021a, 2021b) and Pereira et al. (2022), provide an important basis for evaluating temporal variations in sediment geochemistry within the system. The concentrations of Al, Fe, Cu, Ba (2022), and Co (2022) observed in the present study were higher than those reported by Carneiro et al., (2021a, 2021b), who evaluated the contamination history of the estuary using sediment cores. These differences may reflect temporal and spatial variability in element concentrations within the estuary, as well as variations in sedimentary and hydrodynamic conditions during the sampling periods. In contrast, the concentrations of Ba (2013), As, Cr, and Mn (2013) were lower than those reported by those authors. In contrast, the concentrations of Ba (2013), As, Cr, and Mn (2013) were lower than those reported by those authors.

Pereira et al. (2022), based on surface sediment samples collected in 2014, found higher concentrations of Ba, Zn, Co, Al, and Fe compared to those found in the present study. These differences may reflect spatial and temporal variability in the geochemical composition of estuarine sediments, potentially associated with variations in sediment sources, hydrodynamic and sedimentary conditions, and changes in anthropogenic inputs within the study area. The concentrations of Cr, Cu, Pb, and Zn in the present study are lower than those found by Guo et al. (2019) in intertidal surface sediments of the Yangtze River estuary, China, which is considered polluted (Yi et al., 2021).

Comparing the elemental concentrations in the Serinhaém estuary with the threshold effect level (TEL), probable effect level (PEL), and effects range low (ERL) values (Macdonald et al., 1996), only Ni exceeded the TEL and ERL, suggesting a potential for occasional adverse effects on benthic organisms.

### Polycyclic aromatic hydrocarbons (PAH)

The PAHs are persistent organic pollutants that can have significant deleterious impacts on marine ecosystems, including benthic biota. The determination of PAHs was performed on sediment collected following the 2019 oil spill.

Evaluating the behavior of these compounds, an increase in PAH concentrations is observed toward the upstream portion of the estuary (A1 > A2 > A3 > A4). Individually, among the 16 investigated PAHs, Ace was below the limit of quantification (< LQ) at all sampling points. Acy was found above the LQ only at A1. At A4, only NaP, Flu, Phe, and BaP were not below their respective LQs. Indeed, the only PAHs that did not present concentrations below the LQ at any of the sampled points were NaP, Flu, Phe, and BaP.

NaP is widely identified in environmental matrices, having multiple origins, including smoke emissions and natural combustion of organic matter, as well as activities related to petroleum and its derivatives (Meire et al., 2007). Additionally, it is generated from vehicle emissions, being a recurrent component of automobile exhaust gases (Lia et al., 2024). BaP, in turn, may represent a potential ecological risk in the aquatic environment, affecting various organisms exposed to this contaminant (Qian et al., 2020; Zafarani et al., 2022).

Phe is widely used as an indicator of petrogenic sources (Zafarani et al., 2022) and is frequently associated with urban and industrial effluent discharges (Shen et al., 2024). Exposure to Phe can result in several adverse effects on biota, including bioaccumulation processes in bivalve mollusks, in addition to mutagenic effects already described in the literature (Choueri et al., 2024; Irwin et al., 1997; Yakan et al., 2017). Finger et al. (1985) studied the behavior of organisms exposed to Flu and concluded that it not only represents an immediate threat to certain fish and invertebrates but also has long-term effects that can alter species growth, reproduction, and survival.

Regarding the standard deviation (SD) values among the sampling points presented in Table 7, the differences are considered high for most PAHs. The SD magnitude is very high compared to the mean. For BaA, IP, BghiP, and BbF, the standard deviation is almost as large as the mean, indicating substantial variability among the sampling points. This pattern can be explained, as discussed above, by the significantly lower values observed at station A4, in contrast to the higher concentrations of some PAHs recorded at station A1, which may have been influenced by its proximity to the urban area of Ituberá (BA) and by vessel traffic.

**Table 7:** PAH concentrations (ng g⁻^1^) in surface sediment samples collected from the Serinhaém Estuary in 2022

Points	NaP	Acy	Ace	Flu	Phe	Ant	Fla	Pyr	BaA	Chr	BbF	BkF	BaP	IP	DahA	BghiP
A1	5.09	2.29	< LQ	2.06	10.12	2.19	12.9	9.43	10.93	12.86	15.69	8.87	15.62	19.9	5.38	14.97
A2	5.39	< LQ	< LQ	3.22	12.54	1.98	6.12	5.07	3.66	8.25	6.99	3.99	7.71	10.07	2.42	6.43
A3	1.67	< LQ	< LQ	2.85	13.07	1.37	7.44	5.77	3.71	8.33	7.9	5.18	7.04	7.92	3.08	6.42
A4	1.34	< LQ	< LQ	1.38	8.92	< LQ	< LQ	< LQ	< LQ	< LQ	< LQ	< LQ	2.43	< LQ	< LQ	< LQ
LQ		0.6	0.7	0.5	0.6	0.6	0.6	0.5	0.4	0.6	0.4	0.6	0.6	0.6	0.6	0.6
Mean	3.37	1.02	< LQ	2.38	11.20	1.53	6.55	5.19	4.68	7.51	7.74	4.66	8.2	9.62	2.87	7.11
Min	1.34	< LQ	< LQ	1.38	8.92	< LQ	< LQ	< LQ	< LQ	< LQ	< LQ	< LQ	2.43	< LQ	< LQ	< LQ
Max	5.39	2.29	< LQ	3.22	12.54	2.19	12.9	9.43	10.9	12.86	15.69	8.87	15.62	19.9	5.38	14.97
Std. Dev	2.16	0.85	0	0.82	1.97	0.71	5.0	3.67	4.45	5.09	6.26	3.41	5.48	7.96	1.97	5.92

In contrast, Phe is the only compound where the SD (1.97) is small relative to its mean (11.20), indicating that concentrations of this specific PAH are more consistent across the collection points. This pattern may be explained by the fact that phenanthrene is commonly associated with petroleum-derived inputs, including vessel discharges and fuel-related contamination. Thus, boat traffic throughout the estuary may contribute to its relatively homogeneous distribution among sampling sites. Furthermore, the strong association between Phe and TOC reported by Carneiro et al. (2026) suggests that sedimentary organic matter plays an important role in the retention of this compound, contributing to its more uniform distribution across the estuary.

The highest average concentration among the four collection points was for Phe (11.20 ng g⁻^1^), followed by IP (9.62 ng g⁻^1^). Among the four points (Table 8), A1 presented the highest Σ16PAHs (92.84 ng g⁻^1^), while the lowest concentration was found at A4 (20.87 ng g⁻^1^). Regarding ΣLPAHs, A2 reached the highest concentration (24.43 ng g⁻^1^), while A1 had the lowest (9.44 ng g⁻^1^). For ΣHPAHs, A1 showed the highest concentration (83.40 ng g⁻^1^) and A4 the lowest (7.33 ng g⁻^1^). The average concentrations in the estuary for Σ16PAHs, ΣLPAHs, and ΣHPAHs were 70.48, 16.92, and 53.56, respectively. Assessing the overall concentration, LPAHs represented only 24% of the Σ16PAHs found in the estuary.

**Table 8:** Concentrations (average, minimum, maximum, and standard deviation) of total, low, and high molecular weight PAHs in the Serinhaém estuary

Points	ΣLPAHs	ΣHPAHs	Σ16PAHs
A1	9.44	83.40	92.84
A2	24.43	60.71	85.14
A3	20.26	62.79	83.05
A4	13.54	7.33	20.87
Mean	16.92	53.56	70.48
Min	9.44	7.33	20.87
Max	24.43	83.40	92.84
Std. Dev	6.70	32.48	33.70

Baumard et al. (1998) proposed a classification for PAH contamination levels based on total PAH concentrations (ΣPAHs), establishing four categories: low (0–100 ng g⁻^1^, dry weight), moderate (100–1,000 ng g⁻^1^, dry weight), high (1000–5000 ng g⁻^1^, dry weight), and very high (> 5000 ng g⁻^1^, dry weight). According to this criterion, the Serinhaém estuary falls into a low-to-moderate contamination level.

Environmental quality metrics defined by the Canadian Sediment Quality Guidelines (CSQG) categorize the presence of PAHs in sediments through TEL and PEL levels (Macdonald et al., 1996). In the context of the Serinhaém estuary, surface samples presented maximum levels of ΣLPAHs, ΣHPAHs, and Σ16PAHs of 83.40, 24.43, and 92.84 ng g⁻^1^, respectively. These results are below the limits of 312 ng g⁻^1^ (ΣLPAHs), 655 ng g⁻^1^ (ΣHPAHs), and 1684 ng g⁻^1^ (Σ16PAHs) stipulated for the TEL (Macdonald et al., 1996). Therefore, the assessment conducted two years after the oil spill concludes that surface sediments in the estuary do not contain concentrations reaching the TEL or exceeding the PEL, a level associated with the frequent occurrence of adverse biological impacts (Macdonald et al., 1996). Previous studies conducted in the estuary estimated a sedimentation rate of 0.25 ± 0.01 cm year⁻^1^ (Carneiro et al., 2026). Therefore, the upper 5 cm of sediment analyzed in the present study encompass approximately two decades of sediment accumulation, ensuring that any sediment layer potentially affected by the 2019 oil spill would have been included in the sampled interval.

To identify PAH emission sources, molecular ratios are used to discriminate between petrogenic and pyrogenic processes. For instance, the LMW/HMW ratio associates values > 1.0 with inputs of petroleum and its derivatives, while values < 1.0 are characteristic of combustion processes (Yunker et al., 2002). Regarding the Fla/(Fla + Pyr) ratio, the interpretation is more detailed: indices below 0.4 suggest a petrogenic origin; between 0.4 and 0.5, the predominant source is fossil fuel combustion; and above 0.5, the origin is attributed to the combustion of coal or plant biomass (Yunker et al., 2002; Zhang et al., 2021, 2024). Additionally, the Ant/(Ant + Phe) ratio is used to confirm the source typology, where values above 0.1 signal a pyrogenic origin, contrasting with a petrogenic origin for values below this threshold (Yunker et al., 2002).

Data regarding the diagnostic ratios in the estuary are detailed in Table 9. The analysis of the LMW/HMW ratio revealed that, with the exception of point A4, which showed signs of petrogenic origin, the other sampling points indicated an origin linked to combustion processes. As for the Fla/(Fla + Pyr) ratio, results pointed toward the combustion of plant biomass or coal at all four analyzed points. On the other hand, the Ant/(Ant + Phe) relationship demonstrated a spatial distinction: points A1 and A2 were characterized as having a pyrogenic origin, while A3 and A4 exhibited a petrogenic profile.

**Table 9:** Total PAH concentrations (ng g⁻^1^) and diagnostic ratios in surface sediment samples from the Serinhaém estuary

Points	Ant/Ant + Phe	Fla/Fla + Pyr	LMW/HMW
A1	0.28	0.52	0.11
A2	0.14	0.55	0.40
A3	0.09	0.56	0.32
A4	0.06	0.32	1.94

Carneiro et al. (2026), investigating the history of PAH contamination in the estuary, identified a concentration peak in the central portion of the estuarine channel. The authors hypothesized that this increase may be related to atmospheric inputs associated with historical mining operations in Camamu Bay, although a direct source attribution could not be established. Furthermore, the sediment core collected at the estuary mouth exhibited a predominantly petrogenic signature throughout the profile, whereas only the surface layers could be associated with the 2019 oil spill.

### Structure and diversity of the benthic foraminiferal community

**Fig. 2:**
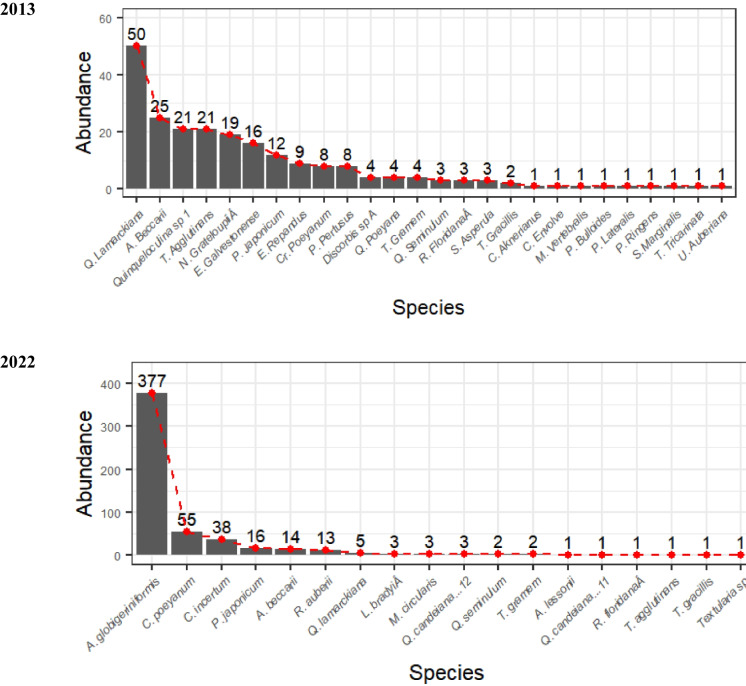
Species abundance of foraminifera by sampling year

In the 2013 campaign, 221 foraminifera (217 in A4, 3 in A3, and 1 in A1) belonging to 19 genera and 26 species were found. Among these, *A. beccarii*, *E. galvestonense*, *N. grateloupii*, *P. japonicum*, *Q. lamarckiana*, *Quinqueloculina* sp. 1, and *T. agglutinans* were dominant (Table 10; Fig. 2).

**Table 10:** Foraminiferal species found in the Serinhaém River estuary during the 2013 and 2022 campaigns

Species	2013				2022			
	A1	A2	A3	A4	A1	A2	A3	A4
*Ammonia beccarii*	*	*	1	24	*	*	7	7
*Ammoglobigerina globigeriniformis*	*	*	*	*	248	32	91	6
*Amphistegina lessonii*	*	*	*	*	*	*	*	1
*Rosalina floridana*	*	*	*	3	*	*	1	*
*Rosalina auberii*	*	*	*	*	*	*	3	10
*Elphidium incertum*	*	*	*	*	*	*	37	1
*Elphidium poeyanum*	*	*	1	7	*	*	46	9
*Miliammina circularis*	*	*	*	*	*	*	1	2
*Pseudononion japonicum*	*	*	*	12	*	*	*	16
*Liebusella bradyi*	*	*	*	*	*	*	*	3
*Quinqueloculina candeiana*	*	*	*	*	*	*	*	4
*Quinqueloculina lamarckiana*	*	*	1	49	*	*	*	5
*Quinqueloculina seminulum*	*	*	*	3	*	*	*	2
*Textularia* sp.	*	*	*	*	*	*	*	1
*Textularia agglutinans*	*	*	*	21	*	*	*	1
*Textularia gramen*	*	*	*	4	*	*	1	1
*Textularia gracilis*	*	*	*	2	*	*	*	1
*Archaias mexicana*	*	*	*	*	1	1	2	*
*Cibicides aknerianus*	1	*	*	*	*	*	*	*
*Cibicides evolutus*	*	*	*	1	*	*	*	*
*Discorbis* sp.* A*	*	*	*	4	*	*	*	*
*Elphidium galvestonense*	*	*	*	16	*	*	*	*
*Eponides repandus*	*	*	*	9	*	*	*	*
*Marginopora vertebralis*	*	*	*	1	*	*	*	*
*Nonionoides grateloupii*	*	*	*	19	*	*	*	*
*Peneroplis pertusus*	*	*	*	8	*	*	*	*
*Puteolina lateralis*	*	*	*	1	*	*	*	*
*Pyrgo bulloides*	*	*	*	1	*	*	*	*
*Pyrgo ringens*	*	*	*	1	*	*	*	*
*Quinqueloculina poeyana*	*	*	*	4	*	*	*	*
*Quinqueloculina* sp.* 1*	*	*	*	21	*	*	*	*
*Spiroloculina asperula*	*	*	*	3	*	*	*	*
*Sorites marginalis*	*	*	*	1	*	*	*	*
*Triloculina tricarinata*	*	*	*	1	*	*	*	*
*Uvigerina auberiana*	*	*	*	1	*	*	*	*
TOTAL	**1**	**0**	**3**	**217**	**249**	**33**	**189**	**70**

*Ammonia* is one of the most abundant and diverse genera of benthic foraminifera. It occurs in marine environments with > 80% mud/silt and is widely found in estuaries and mangroves, as it is capable of dealing with the wide range of salinities, oxygen levels, and temperatures associated with these habitats (Bird et al., 2019), which justifies its predominance in the study area.

The species *Q. lamarckiana* can be found in places with high-energy waters, as it has a resistant test (Machado et al., 2012). This information corroborates the results of the present study, as the presence of this species was most evident in A4, a location with marked hydrodynamics.

In the 2022 campaign, 541 foraminifera (249 in A1, 189 in A3, 70 in A4, and 30 in A2) belonging to 13 genera and 18 species were found. *A. globigeriniformis*, *E. incertum*, and *C. poeyanum* were dominant (Table 10; Fig. 2). Among the species recorded across the four sampling sites, only *Ammoglobigerina globigeriniformis* was classified as a constant species.

*A. globigeriniformis* showed higher abundances at sites with relatively lower pH values, suggesting tolerance to these conditions, although pH exhibited a low coefficient of variation among sampling points (0.034). The species exhibited similar behavior regarding salinity variations, which did not vary significantly between campaigns. Nevertheless, in both campaigns, salinity values decreased upstream as the waters moved away from marine influence, a pattern consistent with that reported by Dong et al. (2020) when investigating the response of benthic foraminiferal communities to pH changes. In studying the zoning of foraminifera in a mangrove forest in the Bertioga Channel (São Paulo, SP, Brazil), Eichler et al. (2007) observed that agglutinating species dominate the mixohaline and brackish sectors of the mangroves, while calcareous forms become more abundant in areas of greater marine influence. The authors highlight those acidic conditions disfavor the construction of calcareous tests, relatively favoring agglutinating species.

Although Eichler et al. (2007) associated *Cribroelphidium poeyanum* with polyhaline environments dominated by sandy sediments, its highest abundance in A3, characterized by 80.87% mud, suggests that grain size was not the primary factor controlling its distribution in the study area. The high water salinity (30.5) and low total organic carbon content (1.79%) observed at this site appear to have favored the occurrence of the species, overriding the influence of sediment type. Ecological studies have demonstrated that the distribution of benthic foraminifera results from the interaction of multiple environmental factors, including salinity, temperature, oxygen availability, food supply, hydrodynamics, and sediment characteristics. In coastal and estuarine environments, however, salinity is frequently considered one of the primary factors controlling the spatial distribution of foraminiferal assemblages (Jorissen et al., 2022).

Alpha diversity, characterized as the diversity within a habitat or sampling unit, is measured by species richness, an intuitive and straightforward metric referring to the number of species observed in a location (Table 11).

**Table 11:** Ecological indices for the 2013 and 2022 campaigns

	Species richness		Species abundance		Margalef index		Shannon–Wiener Index		Pielou Evenness Index	
	2013	2022	2013	2022	2013	2022	2013	2022	2013	2022
A1	1	2	1	249	-	0.18	0	0.0261	-	0
A2	0	2	0	33	0	0.29	0	0.1358	0	0
A3	3	9	3	189	1.82	1.53	1.0986	1.3343	1	0.5000
A4	25	17	217	70	4.46	3.77	2.6015	2.4064	0.8082	0.9182

### Relationships between chemical elements, geochemical parameters, and key biological parameters

**Fig. 3:**
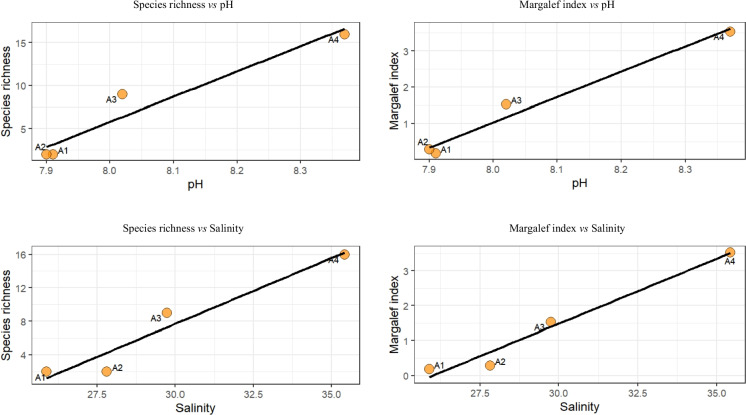
Richness and Margalef index versus pH and salinity in the 2022 campaign

In the 2013 campaign, a positive association was found between species richness and both pH (*F*_1,2_ = 26.818, *p*-value = 0.03532) and salinity (*F*_1,2_ = 29.09, *p-*value = 0.03269). Similarly, the Margalef index was positively associated with pH (*F*_1,2_ = 75.529, *p*-value = 0.01298) and salinity (*F*_1,2_ = 64.944, *p*-value = 0.01505) (Fig. 3). A significant positive association was observed between species richness and both pH and salinity, suggesting that areas under greater marine influence tend to support higher species richness. In contrast, for the 2013 campaign, no significant relationship was observed between species richness or the Margalef index and particle size, physicochemical parameters, or the chemical element concentrations analyzed. The absence of significant associations between species richness, the Margalef index, and the environmental variables assessed in 2013 suggests that community distribution was not predominantly controlled by a single environmental factor. Rather, given the complexity of estuarine environments, species distribution is often driven by the interaction of multiple environmental variables (Murray, 2000).

**Fig. 4:**
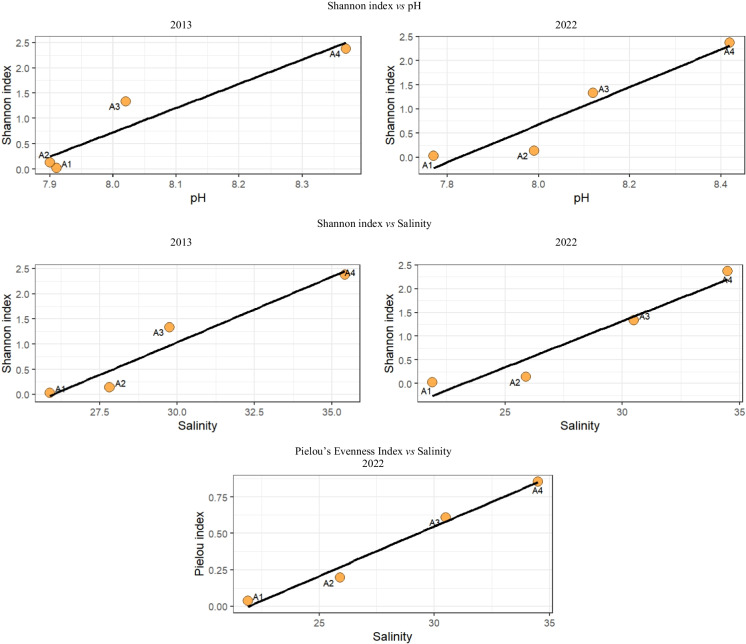
Association between the Shannon Index and Pielou’s Evenness Index with salinity and pH in the two sampling campaigns

Species distribution patterns across the areas were highly discrepant. A4 presented the highest species diversity (Shannon–Wiener H’ = 2.6015 and 2.4064 for 2013 and 2022, respectively), whereas A1 exhibited the lowest diversity (H’ = 0 and 0.0261 for 2013 and 2022, respectively). A similar pattern was observed for evenness in both years; species showed similar abundances (i.e., high evenness) in A4, while the 2022 campaign in A1 was characterized by the dominance of *A. globigeriniformis*, resulting in low evenness (Table 10).

Diversity index Shannon, in the 2013 campaign, a positive association was found between pH (*F*_1,2_ = 18.363, *p*-value = 0.05038) and salinity (*F*_1,2_ = 27.305, *p*-value = 0.03473). Similarly, in 2022, the Shannon index was positively associated with pH (*F*_1,2_ = 18.969, *p*-value = 0.04888) and salinity (*F*_1,2_ = 25.571, *p*-value = 0.03695) (Fig. 3). Pielou index evenness, in 2022, a positive association was found between pH (*F*_1,2_ = 26.551, *p*-value = 0.03566) and salinity (*F*_1,2_ = 108.25, *p*-value = 0.009112).

To better understand these relationships, tests were performed between the dominant species (> 5%) and the physicochemical variables and chemical elements. In 2013, some species showed statistically significant relationships (*p* < 0.05) only with pH (Table 12), likely due to marine influence at site A4. Similar results were observed by Dong et al. (2020), who found a positive association between the abundance of *A. beccarii* and pH in their work carried out in Qingdao Bay, in the Yellow Sea. According to the authors, higher pH conditions favor the preservation and development of its calcareous test, while acidification reduces its abundance. Thus, the higher occurrence of *A. beccarii* in A4 reinforces the hypothesis of marine influence and is consistent with the positive association observed between species abundance and pH, suggesting that this parameter may have influenced its distribution in the study area.

**Table 12:** Association between abundant species in the 2013 campaign and pH

Species	pH	
	*F*	*p*-value
*A. beccarii*	44.092	0.02194
*P. japonicum*	30.797	0.03097
*Q. lamarckiana*	36.411	0.02638
*T. agglutinans*	30.797	0.03097
*E. galvestonense*	30.797	0.03097
*N. grateloupii*	30.797	0.03097
*Quinqueloculina sp 1*	30.797	0.03097

In the 2022 campaign, associations were observed between *A. globigeriniformis* and TOC, as well as some chemical elements (Table 13), suggesting that this taxon may tolerate the environmental conditions associated with these variables. Similar behavior was reported by Balachandar et al. (2023), who investigated benthic foraminifera as environmental proxies for pollutants along the coast of Chennai, India.

**Table 13:** Relationship between abundant species in the 2022 campaign and TOC and selected chemical elements

*Species*	TOC F (*p-*value)	Chemical elements *F* (*p-*value)			
*A_globigeriniformis*	673.67 (0.00148)	Co	Cr	Al	Fe
		57.57 (0.01693)	426.73 (0.002335)	64.69 (0.01511)	95.83 (0.01027)
		Mn	V	Zn	Cu
		31.45 (0.03036)	161,519 (0.0000)	419.97 (0.002343)	306.39 (0.003248)

Regarding the types of foraminifera identified in 2013, hyaline and porcelaneous forms presented equal proportions (44.34% each), totaling 88.68% of the analyzed assemblage, while the remainder corresponded to agglutinated foraminifera. It is worth noting that 98.2% of the foraminifera were identified in sample A4.

In 2022, agglutinated foraminifera predominated (70.94%), followed by hyaline (25.51%) and porcelaneous forms (3.51%). At sites A1 and A2, only agglutinated species were recorded, whereas at sites closer to the downstream region, all three test types were present. Although salinity values remained within the brackish to marine range, the predominance of agglutinated foraminifera at A1 and A2 may also be related to the higher TOC concentrations and lower pH values measured at these sites. Such conditions may promote environmental stress and reduce habitat suitability for calcareous taxa, thereby favoring agglutinated species. This pattern is consistent with previous observations that agglutinated foraminifera are generally more tolerant of variations in salinity and pH. At A3, hyaline and agglutinated taxa were codominant, representing 49.74% and 49.21% of the assemblage, respectively. At A4, hyaline forms predominated (62.86%), followed by porcelaneous (24.29%) and agglutinated forms (12.86%).

In subtropical estuaries, there is generally an ecological succession of marine calcareous foraminifera (present at the mouth), followed by calcareous mixohaline species, which are gradually replaced by agglutinated foraminifera (Duleba et al., 2004). Thus, the predominance of calcareous tests in the 2013 campaign is attributed to the proximity of site A4 to the estuary mouth. However, their near exclusivity suggests that suitable conditions for the survival of these organisms were restricted to this location. In contrast, during the 2022 campaign, estuarine conditions were established at all sites, allowing the survival of a greater number of species and the development of the expected ecological succession of foraminifera.

This behavior may also explain the striking difference in foraminifera abundance between the two sampling campaigns. In 2013, only 221 individuals were recorded, and the assemblage was almost exclusively restricted to station A4. However, 541 individuals were found in 2022, and they were distributed across all sampling stations. The contrasting distribution patterns observed between the two campaigns may be associated with differences in rainfall conditions and related environmental characteristics. Higher rainfall in 2013 may have influenced estuarine conditions differently from those observed in 2022; however, additional sampling would be necessary to better understand the factors associated with these differences.

Taphonomic analysis of the foraminiferal assemblage collected in 2013, based on test color, showed that most of the 221 individuals were white/colorless (48.87%), followed by mottled (22.17%) and yellow specimens (14.93%). Regarding preservation state, most tests were classified as normal (42.53%), followed by dissolved tests (37.10%). The predominance of white tests, together with the relatively high proportion of normally preserved specimens, suggests generally favorable preservation conditions for the assemblage. Nevertheless, the occurrence of dissolved tests indicates that post-depositional alteration processes also affected a substantial portion of the specimens. Therefore, the assemblage appears to record a mixture of well-preserved individuals and specimens subjected to varying degrees of taphonomic alteration.

In the 2022 campaign, a total of 541 individuals were analyzed. Considering all sampling stations together, white tests predominated (75.60%), followed by mottled tests (21.44%). Regarding preservation state, specimens exhibiting multiple types of wear were the most frequent (38.08%), followed by broken tests (36.53%) and normal tests (20.30%). It is important to note that the percentages presented refer to the overall taphonomic condition of the assemblage and do not represent patterns specific to any individual sampling station. According to Moraes and Machado (2003), a mixed preservation pattern may indicate either prolonged residence time in the sediment, resulting in exposure to different alteration processes, or recent deposition combined with multiple degradation mechanisms acting simultaneously. In the present case, the predominance of white tests suggests recent deposition, whereas the occurrence of multiple types of wear may be associated with the test composition of the dominant species, *A. globigeriniformis*, which is agglutinated and therefore more susceptible to breakage and dissolution.

In 2013, only seven specimens containing pyrite were identified, five of which were from site A4. In 2022, 358 out of 541 specimens exhibited pyrite, corresponding to more than 50% of the total. At site A1, 173 out of 249 specimens contained pyrite; at A2, 18 out of 33; at A3, 150 out of 189; and at A4, 17 out of 70. The decomposition of organic matter (OM) by anaerobic bacteria in confined, OM-rich environments can promote pyrite precipitation (Duleba, 1994), which is consistent with the present findings, as dissolved oxygen levels were lower in 2022. Site A1, the furthest from the estuary mouth and characterized by slower water renewal, also exhibited the highest TOC values.

### Relationship between foraminiferal species and PAHs

The influence of PAHs on benthic foraminiferal communities has been widely investigated (Châtelet et al., 2004; Machain-Castillo et al., 2019; Vilela et al., 2011). However, the quantitative assessment of this impact remains a scientific challenge. In PAH-impacted ecosystems, a decline in both population abundance and species diversity of these microorganisms is frequently observed. This pattern results from the sensitivity of various taxa to these contaminants, which compromises their survival and reproductive efficiency under environmental stress conditions (Zhang et al., 2023).

In polluted environments, foraminiferal communities tend to be dominated by a limited number of opportunistic and pollution-tolerant species. These species are able to thrive under conditions that are inhospitable to more sensitive organisms, leading to an overall reduction in species richness (Di Leonardo et al., 2007; Zhang et al., 2023). Furthermore, different groups of foraminifera exhibit varying tolerances to pollutants. For instance, some agglutinated and hyaline species may be favored by the presence of chemical elements, whereas other hyaline and porcelaneous species show greater tolerance to TOC and PAHs. Nevertheless, the prevailing trend is a reduction in species richness due to the toxicity of these compounds (Zhang et al., 2023).

According to Machain-Castillo et al. (2019), critical PAH concentrations exceeding 5000 ng g⁻^1^—substantially higher than those observed in the present study area—can cause severe damage to foraminiferal assemblages. In the context of this study, the detected concentrations did not reach levels high enough to trigger such effects. A similar outcome was reported by Zhang et al. (2023) in their investigation of benthic foraminiferal assemblages in Houshui and Yangpu Bays, on Hainan Island.

To evaluate the relationship between PAHs and foraminifera, a Kruskal-Wallis test was performed. The identified relationships are presented in Table 14. Associations were observed between naphthalene, anthracene, benzo[a]anthracene, and dibenzo[a,h]anthracene and some foraminiferal species (Fig. 4).

**Table 14:** Association between abundant species in the 2022 campaign and PAHs

PAH	Species	*F*	*p*-value
Naphthalene	*A_beccarii*	277.77	0.0036
	*T_gramen*	277.77	0.0036
Anthracene	*R_auberii*	37.98	0.0253
	*M_circularis*	133.27	0.0074
Benzo[a]Anthracene	*A_globigeriniformis*	32.54	0.0294
Dibenzo[ah]Anthracene	*A_globigeriniformis*	20.53	0.0454

**Fig. 5:**
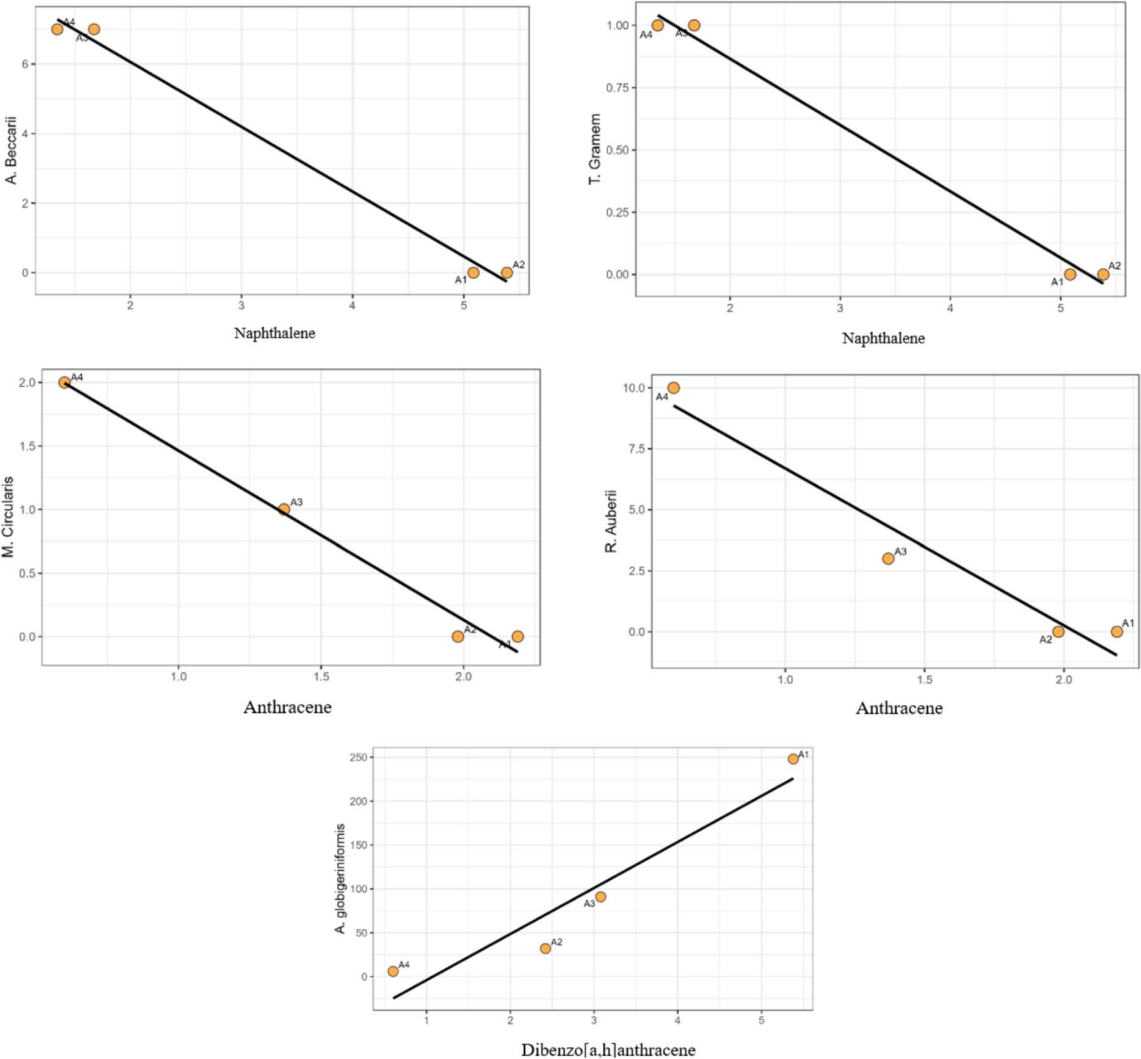
Associations between PAHs (naphthalene, anthracene, and dibenzo[a,h]anthracene) and foraminiferal species

Naphthalene showed a negative association with *A. beccarii* (*F* = 277.77, *p* = 0.0036) and *Textularia gramen* (*F* = 277.77, *p* = 0.0036) (Fig. 5). Species of the genus Ammonia, such as *A. beccarii*, are widely recognized in the literature as tolerant to environmental stressors, including chemical elements and PAHs, and often dominate polluted environments (Di Leonardo et al., 2007; Zhang et al., 2023). However, in the present study, both species exhibited a negative association with naphthalene, suggesting that reductions in their abundance may be linked to increased bioavailability of this compound. LMW PAHs, such as naphthalene, have higher solubility and, consequently, greater interaction with benthic biota, potentially impairing physiological processes and reducing organism abundance (Vijayan et al., 2023; Yunker et al., 2002).

A similar pattern was observed for anthracene (Fig. 5). Negative associations were found between this compound and the species *Miliammina circularis* and *Reophax auberi*. However, considering the low abundance of these species, these results should be interpreted with caution. In addition to the presence of PAHs, other environmental factors, such as sedimentary characteristics, TOC, salinity, and local physicochemical conditions, such as pH, may have contributed to the observed variability in the distribution of these species. HMW PAHs exhibit lower solubility and greater affinity for sediment particles, leading to preferential accumulation in this compartment (Achten & Andersson, 2015; Yunker et al., 2002). Since benthic foraminifera inhabit the sediment–water interface, their distribution may be directly affected by contaminants associated with sediments.

In contrast, dibenzo[a,h]anthracene showed a positive association with *A. globigeriniformis* (Fig. 5). The occurrence of *A. globigeriniformis* in high abundance in areas with elevated dibenzo[a,h]anthracene concentrations suggest a differentiated response of this species to contamination. This may indicate that the species are capable of tolerating or even thriving under such conditions, or that this compound is not lethal at the observed concentrations.

**Fig. 6:**
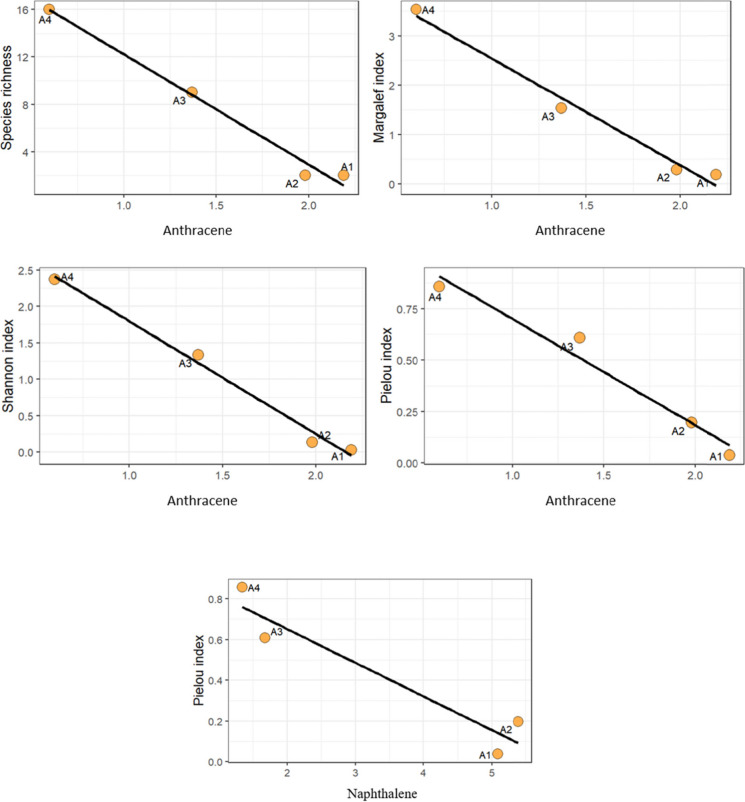
Association between benthic foraminiferal species richness in the Serinhaém estuary and anthracene and naphthalene

The same approach was applied to evaluate the relationship between PAHs and foraminiferal diversity indices. The results (Fig. 6) indicate a negative relationship between anthracene concentration and benthic foraminiferal species richness (*F*₁,₂ = 133.27, *p* = 0.007). A similar trend was observed for the Margalef index (*F*₁,₂ = 116.94, *p* = 0.0084), Shannon index (*F*₁,₂ = 183.89, *p* = 0.0054), and Pielou’s evenness index (*F*₁,₂ = 56.29, *p* = 0.0173). These results suggest that increasing anthracene levels are associated with reduced diversity of benthic foraminiferal assemblages. Naphthalene showed a similar pattern with Pielou’s index (*F*₁,₂ = 18.82, *p* = 0.0493).

Although Zhang et al. (2023) suggested that the effects of chemical elements and TOC may be more pronounced than those of PAHs in some environments, they still recognize the impact of PAHs on foraminiferal communities. The observed decrease in species richness is consistent with the understanding that anthracene, as an environmental stressor, selectively affects less tolerant species, resulting in less diverse assemblages.

Di Leonardo et al. (2007) reported that increasing total PAH concentrations are associated with higher frequencies of morphological deformities and reduced population density in benthic foraminifera. Additionally, they noted that higher contamination levels are often accompanied by an increase in opportunistic, pollution-tolerant taxa. The combined chemical and biological patterns observed in the present study suggest that environmental contamination exerts a restrictive effect on population density and microfaunal structure.

Considering that PAH concentrations below 10 mg kg⁻^1^ (10,000 ng g⁻^1^) have been associated with mutagenic effects (Vondraček et al., 2001), the sediments analyzed in this study showed values significantly lower than this threshold, with maximum concentrations on the order of 10^2^ ng g⁻^1^. These results indicate a low genotoxic potential according to this criterion, although sublethal effects associated with chronic exposure cannot be completely ruled out.

### Relationship between chemical elements and PAHs

The relationship between chemical elements and PAHs was also evaluated, and significant positive associations (*p* < 0.05) were observed exclusively for HMW PAHs, such as benzo[a]anthracene, benzo[k]fluoranthene, benzo[a]pyrene, dibenzo[a,h]anthracene, and benzo[ghi]perylene (Table 15). All associations were positive, indicating that the concentrations of both contaminants exhibit similar behavior or are associated by the same depositional processes.

**Table 15:** Relationship between chemical elements and PAHs in the 2022 campaign

Chemical elements	Benzo[a]antraceno	
	F	*p*-value
*Co*	20.21	0.0461
*Cr*	20.57	0.0453
*Ni*	19.59	0.0475
*V*	33.27	0.0288
*Zn*	48.08	0.0202
*Cu*	26.78	0.0354
	**Benzo[k]fluoranteno**	
*Al*	25.21	0.0375
*Fe*	19.27	0.0482
*Cu*	21.91	0.0427
	**Benzo[a]pireno**	
*Zn*	21.07	0.0443
	**Dibenzo[ah]antraceno**	
*Al*	30.65	0.0311
*Fe*	23.61	0.0398
*V*	21.04	0.0444
*Zn*	18.74	0.0494
*Cu*	28.88	0.0329
	**Benzo[ghi]perileno**	
*Zn*	20.74	0.045
*Cu*	18.93	0.049

**Fig. 7:**
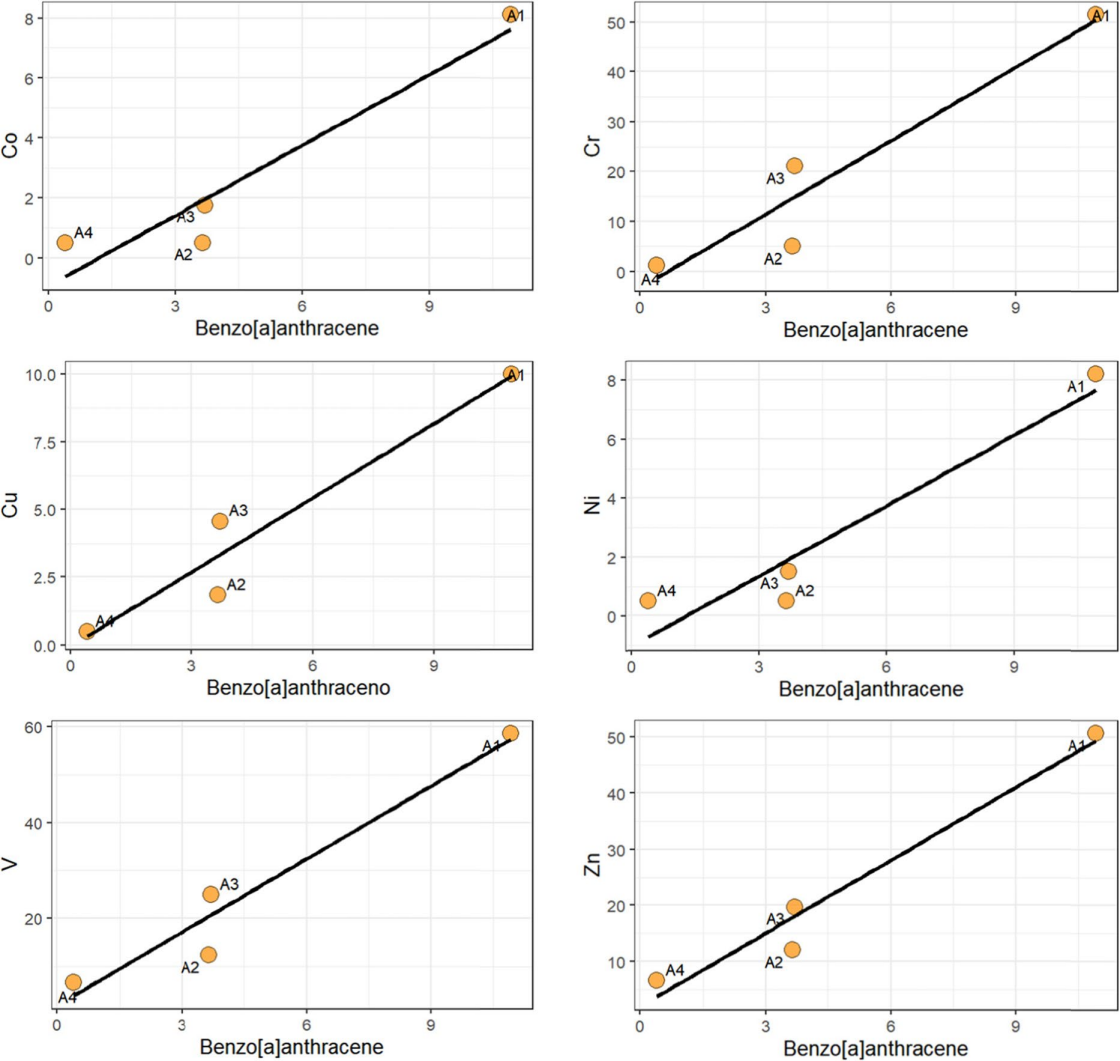
Association between chemical elements and benzo[a]anthracene

**Fig. 8:**
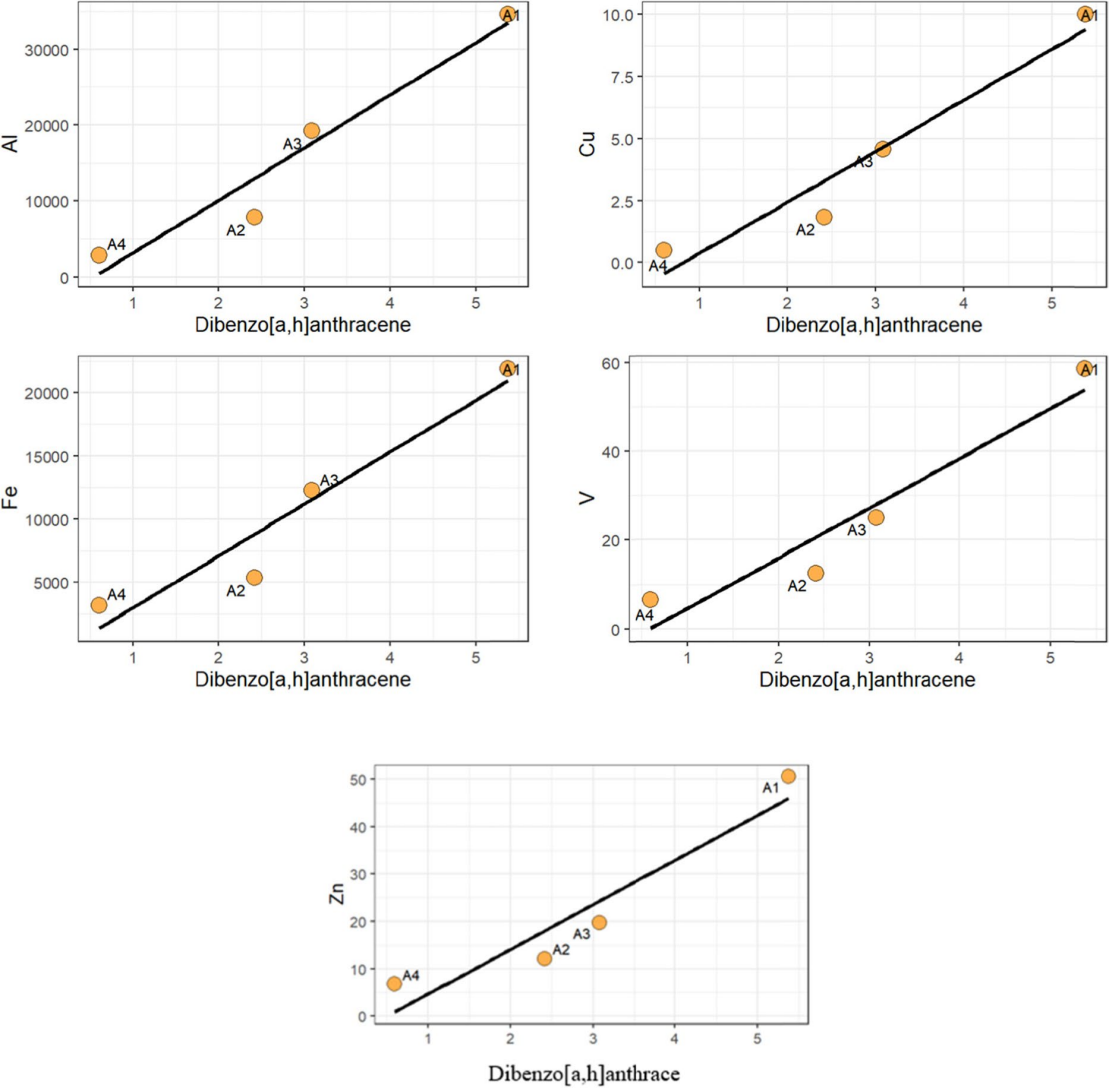
Association between chemical elements and dibenzo[a,h]anthracene

**Fig. 9:**
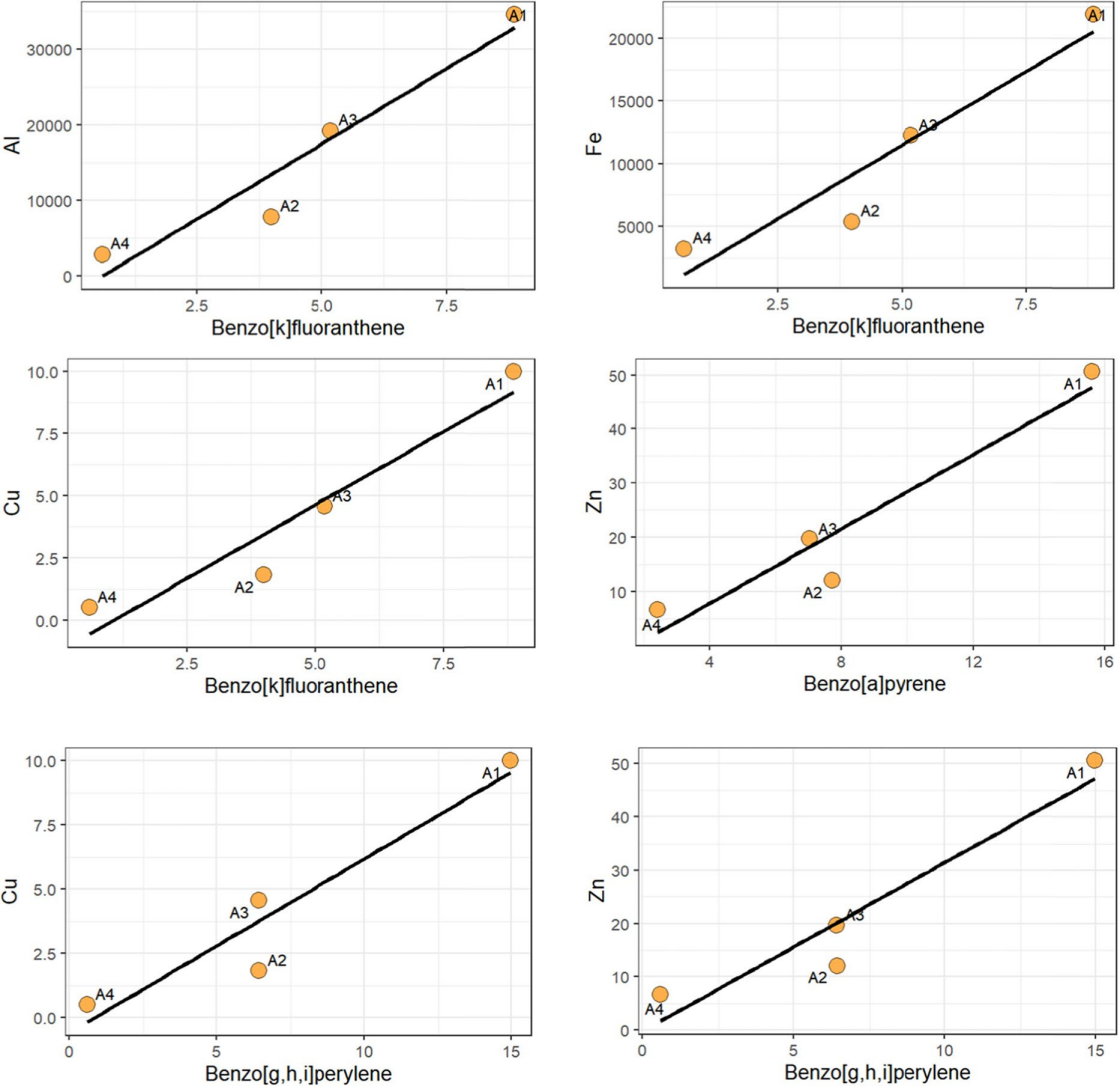
Association between chemical elements and PAHs: (Al, Fe, and Cu) vs. Benzo[k]fluoranthene; Zn vs. Benzo[a]pyrene; and Cu and Zn vs. Benzo[ghi]perylene

Figures 7, 8, and 9 illustrate the relationships between PAHs and the analyzed chemical elements. Among these elements, Cu and Zn showed associations with most PAHs, suggesting a common source. The strong association with Zn and Cu, combined with the predominance of HMW PAHs, indicates a predominantly pyrolitic influence related to anthropogenic activities, such as fossil fuel combustion and biomass burning (Yunker et al., 2002).

The association with V and Ni suggests a possible additional contribution from petrogenic or mixed sources. These elements are commonly found in petroleum and its derivatives and are widely used as geochemical indicators in environmental studies (Souza et al., 2006). The simultaneous presence of elements associated with both combustion processes and petroleum-derived inputs therefore suggests the influence of multiple contamination sources in the study area.

A similar positive association between PAHs and chemical elements was reported in snow samples from northeastern Bavaria (Herrmann, 1987), and it is well established that atmospheric particles enriched in chemical elements also tend to be enriched in PAHs. Consequently, increased coal combustion is expected to elevate PAH and chemical element levels in the environment as a whole, not only in sediments.

Similarly, Forstner and Muller (1981), in their study of chemical element and PAH concentrations in fluvial sediments from Trier, West Germany, also reported positive associations between these contaminants. The authors emphasized that atmospheric particles with high concentrations of chemical elements tend to be enriched in PAHs, indicating that atmospheric deposition represents an important pathway for the input of these contaminants, contributing to their accumulation in different environmental compartments.

The statistical analyses performed in this study were intended to identify potential associations between environmental variables and foraminiferal assemblage attributes. Given the limited number of sampling stations and sampling campaigns, these analyses should be interpreted as exploratory rather than confirmatory. Consequently, the observed associations indicate possible ecological relationships that warrant further investigation through expanded temporal and spatial monitoring.

## Conclusion

The integrated environmental assessment of the Serinhaém River estuary demonstrates that, with respect to chemical elements, the area (located within the Pratigi Environmental Protection Area) is preserved. No significant differences were detected in total chemical elements concentrations between the periods of 2013 (pre‑incident) and 2022 (post‑oil spill). The spatial distribution of these elements along the estuary is governed primarily by natural factors, being associated with the presence of iron and manganese oxyhydroxides and aluminosilicates.

The population dynamics of benthic foraminifera varied according to the hydrological conditions of each period. In 2013, the results suggest that the high river flow may have contributed by rainfall may have contributed to the transport of communities towards the estuary mouth, while in 2022, under conditions of lower precipitation and more confined waters, salinity and pH were probably among the main factors associated with the distribution of microfauna. However, considering the reduced number of sampling campaigns carried out throughout the analyzed period, these patterns should be interpreted with caution and not necessarily as indicative of long-term environmental changes.

Overall, species distribution was not impacted by local chemical element concentrations, with the exception of *Ammoglobigerina globigeriniformis*, which dominated the innermost region of the estuary and showed a strong positive association with TOC and several chemical elements, evidencing its high tolerance.

Regarding the assessment of PAHs carried out in 2022, the estuary exhibited contamination levels considered low to moderate, not exceeding the safety thresholds associated with frequent adverse biological impacts. Diagnostic ratio analyses revealed that these compounds are predominantly pyrogenic in origin, although petrogenic signatures were also occasionally identified, pointing to a scenario of mixed sources encompassing both anthropogenic atmospheric deposition and potential oil residues.

Although concentrations did not reach acute toxicity levels, data integration confirmed that PAHs act as important environmental stressors in the estuary. The presence of compounds such as anthracene and naphthalene associates negatively with overall foraminifera species richness and diversity, and also negatively impacted the abundance of specific taxa. In contrast, a clear process of ecological selection was observed, in which more sensitive species decline while opportunistic and tolerant species survive and proliferate.

In summary, the results reinforce the viability and remarkable sensitivity of benthic foraminifera as robust bioindicators. They are capable of recording early changes in ecological community structure in response to the presence of hydrocarbons and other environmental variables, proving to be an essential tool for the continuous monitoring of environmental quality in sensitive coastal ecosystems.

## Data Availability

No datasets were generated or analysed during the current study.
